# Engineering bone/cartilage organoids: strategy, progress, and application

**DOI:** 10.1038/s41413-024-00376-y

**Published:** 2024-11-20

**Authors:** Long Bai, Dongyang Zhou, Guangfeng Li, Jinlong Liu, Xiao Chen, Jiacan Su

**Affiliations:** 1 Department of Orthopedics, Xinhua Hospital Affiliated to Shanghai Jiao Tong University School of Medicine, Shanghai, China; 2 Organoid Research Center, Institute of Translational Medicine, Shanghai University, Shanghai, China; 3 National Center for Translational Medicine (Shanghai) SHU Branch, Shanghai University, Shanghai, China; 4 Wenzhou Institute of Shanghai University, Wenzhou, Zhejiang China; 5 Department of Orthopedics, Shanghai Zhongye Hospital, Shanghai, China

**Keywords:** Bone, Bone quality and biomechanics

## Abstract

The concept and development of bone/cartilage organoids are rapidly gaining momentum, providing opportunities for both fundamental and translational research in bone biology. Bone/cartilage organoids, essentially miniature bone/cartilage tissues grown in vitro, enable the study of complex cellular interactions, biological processes, and disease pathology in a representative and controlled environment. This review provides a comprehensive and up-to-date overview of the field, focusing on the strategies for bone/cartilage organoid construction strategies, progresses in the research, and potential applications. We delve into the significance of selecting appropriate cells, matrix gels, cytokines/inducers, and construction techniques. Moreover, we explore the role of bone/cartilage organoids in advancing our understanding of bone/cartilage reconstruction, disease modeling, drug screening, disease prevention, and treatment strategies. While acknowledging the potential of these organoids, we discuss the inherent challenges and limitations in the field and propose potential solutions, including the use of bioprinting for organoid induction, AI for improved screening processes, and the exploration of assembloids for more complex, multicellular bone/cartilage organoids models. We believe that with continuous refinement and standardization, bone/cartilage organoids can profoundly impact patient-specific therapeutic interventions and lead the way in regenerative medicine.

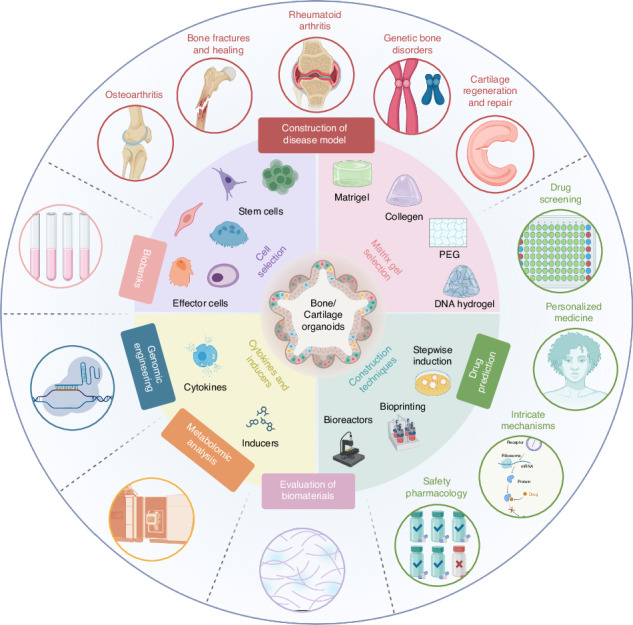

## Introduction

As integral constituents of the musculoskeletal system, bone and cartilage are pivotal in facilitating locomotion, providing structural integrity, and performing diverse physiological functions.^1^ Nevertheless, the prevalence of degenerative and traumatic conditions that compromise these tissues, including osteoporosis, osteoarthritis, and severe bone defects, has been escalating annually in tandem with the progression of population aging and elongation of life expectancy. Such disorders present formidable clinical challenges, substantially diminishing patient quality of life and exerting significant strain on global healthcare infrastructures.^2^ Indeed, the convoluted cellular mechanisms that preside over these degenerative processes remain largely elusive, posing impediments to the development of efficient treatment regimens. Despite the significant advancements of traditional tissue engineering methodologies, they are invariably plagued by various complications such as donor site morbidity, potential for graft rejection, and inadequate restoration of the original tissue functionality.^3^ Moreover, the nuanced intricacies and diverse cellular constitution intrinsic to native bone and cartilage, which underpin their indispensable functions, often prove challenging for these conventional strategies to accurately emulate.^4^ This degree of complexity, when combined with the inherent shortcomings of conventional two-dimensional (2D) cell culture systems, further obscures our comprehension of disease pathogenesis and hinders the evolution of efficacious therapeutic interventions.^5^

**Fig. 1:**
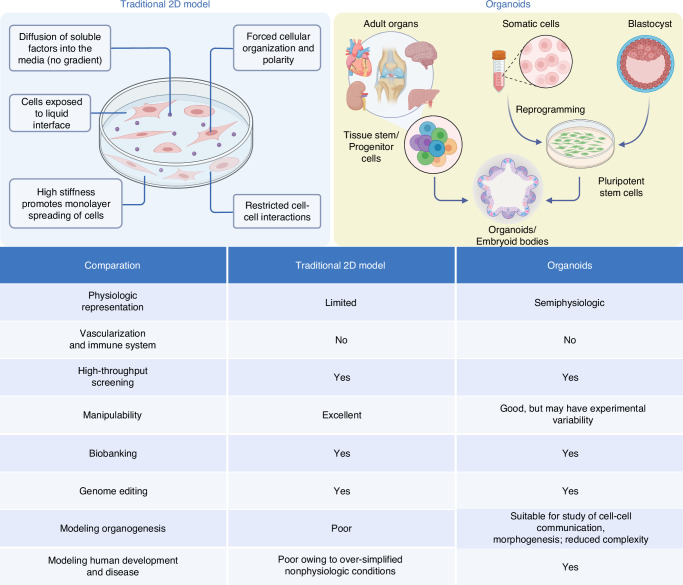
Comparation of traditional 2D models and organoids. The figure compares traditional 2D cell cultures with organoids. The 2D model shows limited physiological relevance, forced cell polarity, and restricted cell interactions, making it ideal for high-throughput screening but inadequate for complex biological processes. In contrast, organoids, derived from pluripotent stem cells or progenitor cells, offer semi-physiological structures, enabling the study of tissue morphogenesis and human development. The table highlights key differences: organoids excel in modeling complex systems but have higher experimental variability, while 2D models remain highly manipulable and suitable for basic screenings

Organoids, miniature, self-organized three-dimensional tissue cultures, have emerged as a promising approach to overcome these limitations.^6^ Derived from both stem cells and progenitor cells, organoids can partially mirror the architecture and functionality of the organ from which they are derived, thereby offering a more physiologically relevant system. However, it is important to note that organoids do not always fully recapitulate the spatial organization of the original organ, and current organoid models often fall short in replicating the full complexity and functionality of mature tissues. While these models provide valuable insights, their ability to faithfully mimic the intricate vascularization, mechanical properties, and long-term functionality of bone and cartilage remains limited. This unique ability to capture the complex spatial and cell-type specific interactions of an organ makes organoids an attractive tool for studying tissue development, disease pathogenesis, and drug responses in a controlled, reproducible manner.^7^ Among these, bone and cartilage organoids stand out as particularly relevant for addressing the previously mentioned challenges. They offer a solution to many of the limitations of traditional tissue engineering and 2D cell culture models (Fig. 1). As three-dimensional structures that mimic the complexity and cell diversity of bone and cartilage tissues, they provide a more representative model for studying the mechanisms of bone and cartilage diseases, as well as testing potential treatments.^8^ Furthermore, bone and cartilage organoids have the potential to serve as innovative tools in regenerative medicine, potentially offering personalized therapeutic options for bone and cartilage repair and replacement.^9^ Nonetheless, the current generation of bone and cartilage organoids still faces challenges in achieving full functional equivalence with natural tissues, particularly in terms of mechanical strength and integration with host tissues. In addition, their use in high-throughput drug screening could accelerate the development of new therapeutics and provide individualized treatment options.^10^ Therefore, the development and refinement of bone and cartilage organoids hold substantial promise for advancing our understanding of bone and cartilage biology and significantly improving patient-specific therapeutic strategies.

Despite the promising potential of bone and cartilage organoids, it is important to acknowledge that this research area is still in its nascent stages with numerous challenges yet to be addressed. For instance, replicating the intricate cellular composition, architecture, and biomechanical properties of native bone and cartilage remains a significant hurdle.^11^ One of the most daunting challenges is achieving proper vascularization in bone organoids, as the formation of a functional vascular network is critical for nutrient delivery and waste removal, which are essential for the viability and maturation of bone tissue. Standardization of protocols for the generation of organoids, maintaining their long-term stability, and validating their predictability and reliability for clinical applications are additional challenges that need to be tackled.^12^ It is against this backdrop of exciting potential, yet substantial challenges, that this review is positioned. Our aim is to provide a comprehensive overview of the current state of the field, including the strategies for constructing bone and cartilage organoids, the progress achieved thus far, and potential applications. Additionally, we seek to outline the current limitations and propose future directions to further advance this field. Through this review, we hope to foster a deeper understanding of bone and cartilage organoids, and inspire continued efforts towards their refinement and application in both basic research and translational medicine.

## Cell microenvironment in bone/cartilage repair

**Fig. 2:**
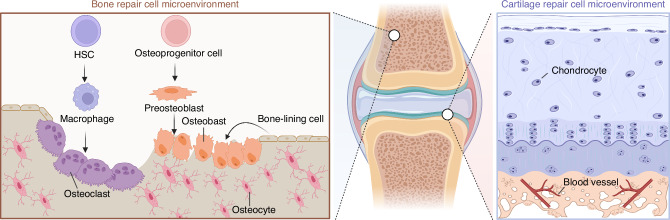
Cell microenvironment in bone/cartilage repair. The figure illustrates the distinct cellular microenvironments involved in bone and cartilage repair. On the left, the bone repair microenvironment consists of hematopoietic stem cells (HSCs), osteoprogenitor cells, and macrophages, which differentiate into osteoclasts for bone resorption, and osteoblasts for bone formation. Osteoblasts mature into osteocytes and bone-lining cells, contributing to bone homeostasis. On the right, the cartilage repair microenvironment is dominated by chondrocytes, which are responsible for cartilage matrix production. The vascularized subchondral layer supports nutrient exchange, crucial for cartilage repair and regeneration. The central image highlights the anatomical location of bone and cartilage within a joint

In the process of bone and cartilage repair, the synergistic action of various cell types is crucial (Fig. 2). During bone repair, hematopoietic stem cells (HSCs) lay the foundation for the immune system, providing essential immune cells like macrophages, which clear dead cells and debris in the early stages of injury and promote the recruitment and activation of other cells by secreting growth factors. Osteoprogenitor cells, stem and progenitor cells found in bone marrow and other bony tissues, can differentiate into pre-osteoblasts, the direct precursors to osteoblasts responsible for synthesizing and mineralizing bone matrix, thus forming new bone. Osteoblasts transform into bone-lining cells once the new bone is fully mineralized, covering the bone surface in a non-active state to regulate bone metabolism and calcium ion balance. Osteoclasts, large multinucleated cells, absorb and remodel bone by secreting acidic substances to dissolve the bone matrix, creating space for new bone formation. The dynamic balance between osteoclasts and osteoblasts is key to maintaining bone health and effective repair. Hematopoietic stem/progenitor cells are crucial for bone resorption as they differentiate into osteoclasts, while osteoblasts arise from skeletal stem/progenitor cells, which are subsets of bone marrow stromal cells, periosteal skeletal stem cells, or growth plate skeletal stem cells. These skeletal stem cells contribute to cartilage and bone formation in various contexts, with periosteal SSCs producing cartilage primarily after injury. Additionally, depending on the location of the source of the cells, such as the periosteum or the bone marrow, they can also differentiate into chondrocytes and form cartilaginous tissues. Mature osteocytes, evolved from osteoblasts, are the primary cell type in bone tissue, embedded within the bone matrix. They form a network with surrounding osteoblasts, bone-lining cells, and other bone cells through their cellular processes, regulating bone metabolism and remodeling.

As a crucial component of the cartilage repair microenvironment, chondrocytes play a pivotal role in the regeneration process. They synthesize and secrete extracellular matrix (ECM) components such as collagen type II and proteoglycans, which are crucial for maintaining cartilage structure and function. The implantation of allogeneic chondrocytes into cartilage defects can enhance the repair process by integrating with the host tissue, promoting ECM production, and improving the biomechanical properties of the regenerated cartilage. Additionally, chondrocytes release anti-inflammatory cytokines and growth factors that support tissue repair and modulate the local microenvironment, further facilitating cartilage regeneration and healing. However, it is important to note that chondrocytes inherently possess limited intrinsic repair capacity. Nevertheless, under certain conditions, inducing these cells to secrete reparative factors or actively participate in the repair process could be beneficial, thereby enhancing their contribution to cartilage regeneration. This cellular activity underscores the importance of chondrocytes in developing effective therapeutic strategies for cartilage repair.

## Introduction of organoids and development of bone/cartilage organoids

The term “organoids” is derived from the combination of the word “organ”—a part of an organism that is typically self-contained and has a specific vital function, such as the heart or liver in humans, and the suffix “-oid”, which comes from the ancient Greek word for “similar” or “resembling”.^13^ The term “organoid” thus primarily emphasizes the resemblance of these structures to actual organs in terms of form and function. Organoids are three-dimensional cellular constructs that mimic the properties of organs in living organisms. They are typically derived from stem cells, which self-organize into structures that closely resemble their corresponding native organs in both form and function.^14^ Although these organoids cannot be considered as true human organs, they have highly similar structures and functions to real human organs and can be stably passaged in vitro. However, it is important to acknowledge that while organoids represent a significant advancement in replicating tissue structures and functions, they still cannot be regarded as true organs. We recognize that achieving organoids with structures and functions that closely mirror those of natural organs remains an aspirational goal. Over the past decade, the development of organoids has been hailed as one of the most exciting advances in stem cell research.^15^ The term “organoid” was proposed as early as 1907,^16^ but it was not until 2009^17^ that Hans Clevers and his team first cultured intestinal stem cells in vitro into a three-dimensional structure with crypt-like and villus-like epithelial regions, namely small intestinal organoids, which marked the beginning of organoid research. Since then, organoid research has entered a period of rapid development. In 2011,^18^ researchers successfully cultivated gut organoids from both human pluripotent stem cells and primary adult stem cells in vitro, and also managed to grow retinal organoids from mouse embryonic stem cells for the first time.^19^ Progress continued into 2012, with the successful cultivation of retinal organoids from human pluripotent stem cells.^20^ The following year marked another milestone with the successful cultivation of brain organoids, along with liver, kidney, and pancreas organoids, all derived from human pluripotent stem cells.^21^ In 2015, mammary gland, fallopian tube, and hippocampus organoids were grown successfully.^22–24^ In 2020, researchers achieved a breakthrough by successfully cultivating skin organoids which can be used to reconstitute skin in vivo.^25^

Research on bone and cartilage organoids, unlike other organoid types, remains in its infancy. The research on osteochondral organoids is in its early stages, with stem cells playing a crucial role in their construction. Stem cells are vital due to their ability to differentiate into various cell types, including those forming bone and cartilage, which are essential for mimicking the complex structure of osteochondral tissues. By introducing stem cells, the goal is to develop bone and cartilage organoids that replicate the structural, functional, and biochemical properties of native bone and cartilage, facilitating studies on disease mechanisms, drug testing, and regenerative medicine applications. A self-structuring model of bone formation using femoral periosteal cells in fibrin gel with calcium phosphate ceramic anchors simulates mature bone chemistry and structure.^26^ Over a year, the model shows collagen-organized hydroxyapatite (HYP) mineral and viable osteocyte-linked canaliculi. Subsequent studies, led by Akiva A., Park, O’Connor, and Hall, successfully generated bone organoids simulating bone formation using various cell sources, including bone marrow mesenchymal stem cells (BMSCs), induced pluripotent stem cells (iPSCs), human periosteum-derived cells (hPDCs).^27–30^ Ultimately, the use of stem cells aims to create osteochondral organoids that not only replicate the native architecture but also possess functional attributes such as load-bearing capacity and integration with host tissues. This approach holds promise for advancing regenerative therapies for osteochondral defects, providing more accurate models for studying osteoarthritis and other joint diseases, and enabling high-throughput screening of potential therapeutic agents.

In 2023, there have been some breakthrough advancements in bone/cartilage organoids. Ouyang et al. reported a study on the development of cartilage organoids through the programmatic introduction of specific growth and developmental signals in the culture system, achieving effective regulation of the proliferation and regenerative potential of human chondrocytes. However, the study did not evaluate the expression of type X collagen, a key marker of hypertrophy, which is critical to confirming the stability of the cartilage formed.^31^ This has resulted in the production of homogeneous cartilage tissue with a diameter of 3.25 mm in an in vitro environment without the participation of biomaterial scaffolds. This size surpasses the current common size (1.5 mm diameter), providing a high-quality ready-to-use transplant for the treatment of joint cartilage damage. It also provides new strategies and technologies for cartilage regeneration. Meanwhile, Yanan Du et al. presented a cutting-edge approach to osteochondral tissue regeneration using in situ self-assembled organoids with dual functional units, which consist of a cartilage layer and a bone layer, and integrated into a single hierarchical structure. The resulting organoids exhibit excellent mechanical properties and biocompatibility, making them a promising candidate for osteochondral tissue regeneration.^32^

As we advance in our understanding and capability to generate bone/cartilage organoids, the potential of assembloids as the next frontier in organoid research is becoming evident. Assembloids are advanced 3D cell culture systems that integrate multiple organoids or specialized cell types to better mimic the complexity of native tissues. By combining different organoids, assembloids can create more physiologically relevant models that incorporate the cellular diversity and spatial organization found in vivo.^33^ One of the potential advantages of assembloids is their ability to model tissue-tissue interactions more accurately than traditional single-tissue organoids. This capability allows researchers to investigate the molecular and cellular mechanisms underlying tissue development, disease progression, and tissue repair in a more integrated and holistic manner. Moreover, assembloids can overcome several limitations associated with current organoid technology. Traditional organoids often face challenges in replicating the full complexity of native tissues due to their limited cellular diversity and lack of organized tissue architecture.^34^ Assembloids address these issues by incorporating multiple cell types and creating spatially organized structures that better resemble the native tissue environment. This enhanced complexity makes assembloids particularly useful for drug screening and disease modeling, providing a more accurate platform for testing therapeutic interventions.^35^ Additionally, the use of assembloids can improve the long-term viability and functionality of organoid cultures. By mimicking the natural tissue environment more closely, assembloids can support better nutrient diffusion and waste removal, reducing issues such as necrosis that are common in traditional organoids.^36^ Overall, assembloids, being 3D cell culture systems that integrate various organoids or specialized cell types, could offer an enhanced level of complexity and functionality, potentially overcoming current limitations in bone and cartilage organoid technology.^33,37–39^

**Fig. 3:**
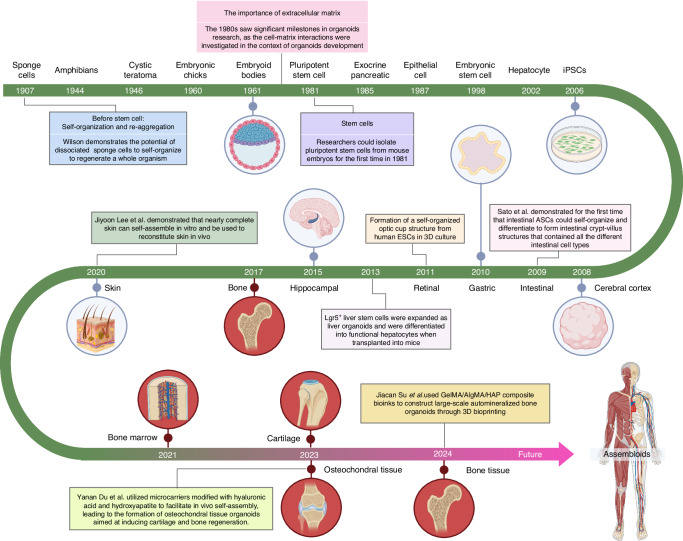
A brief history of organoids and development of bone/cartilage organoids. The diagram outlines the key milestones in organoid research, beginning with the discovery in 1907 that dissociated sponge cells could self-organize, a foundational concept in this field. Over the decades, advances included the development of embryoid bodies (1960), the isolation of pluripotent stem cells (1981), and breakthroughs in generating organ-specific organoids such as intestinal (2009) and liver organoids (2013). The timeline also highlights the role of the extracellular matrix (1980s) in organoid development. Recent advances are marked by the generation of bone and cartilage organoids (2021), and the creation of osteochondral tissue using microcarriers and 3D bioprinting (2023). By 2024, Jiacan et al. employed a GelMA/AlgMA/HAP composite bioink to 3D bioprint large-scale biomineralized bone organoids, marking a significant leap in bone tissue engineering. The future of organoid technology points toward assembloids, which integrate multiple organoid types to mimic complex tissue interactions, offering potential for more sophisticated biological models and therapeutic applications

Applying the concept of assembloids to bone/cartilage research might pave the way for mimicking the intricate cellular and molecular interactions of these tissues more accurately. For instance, the assembly of osteoblasts and osteoclasts in a bone organoid could be further integrated with chondrocytes in a cartilage organoid to form a bone-cartilage assembloid. Such a structure could more realistically model the osteochondral interface, a critical component in joint physiology and pathology. One of the key challenges in developing these complex structures is ensuring adequate nutrient diffusion and waste removal. By incorporating vascular cells, assembloids may overcome these limitations through the formation of capillary-like networks that facilitate nutrient delivery and waste clearance, closely mimicking the natural vascularization of bone and cartilage tissues. Moreover, the incorporation of other cell types, such as vascular or neural cells, into bone/cartilage assembloids could help replicate the important interactions between these tissues and the surrounding vasculature and innervation. Multi-lineage assembloids could also be utilized to study the effects of diseases that affect multiple cell types within the bone or cartilage, such as osteoarthritis or rheumatoid arthritis (RA). The advent of inter-individual and inter-species assembloids could further revolutionize the field by providing novel models to study cell-autonomous effects or to make comparative studies between different species, respectively. However, vascularization remains a significant challenge, especially for larger constructs, and achieving functional blood vessel networks within assembloids is essential for their long-term viability and functionality. Inter-species assembloids are essential for comparative studies that can reveal both conserved and divergent mechanisms across different species. These assembloids enable researchers to identify universal therapeutic targets by comparing human cells with those of other species, thus enhancing the translatability of research findings from animal models to humans. This approach is crucial for validating findings in a broader biological context and for developing treatments that are effective across different biological systems. In conclusion, while the application of assembloids to bone/cartilage research is still largely unexplored, the potential of this approach to enhance our understanding of bone/cartilage biology, and to develop more effective therapeutic strategies, is immense. Future research should not only focus on the integration of specialized cell types and tissues within assembloids but also on overcoming the challenges associated with nutrient diffusion, waste removal, and vascularization. This will be critical in validating the functionality and reliability of the resulting assembloids (Fig. 3).

## Construction strategies of bone/cartilage organoids

**Fig. 4:**
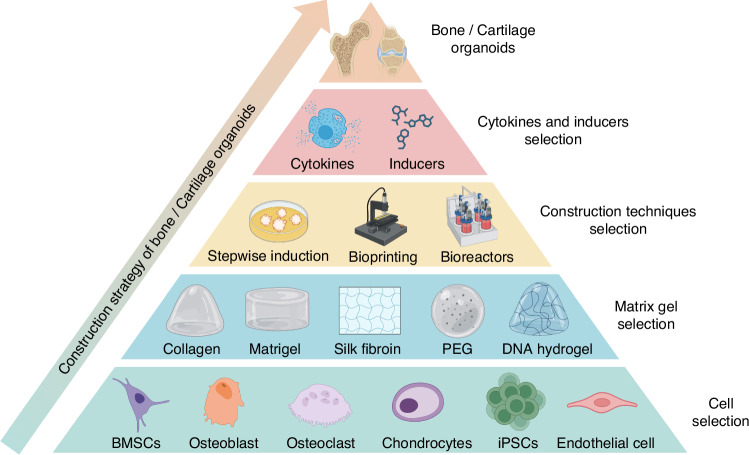
The construction strategy of bone and cartilage organoids. The pyramid outlines the stepwise strategy for constructing bone and cartilage organoids, starting from the base and progressing to the top. Cell Selection: The foundation of the strategy includes choosing appropriate cell types, such as BMSCs, osteoblasts, osteoclasts, chondrocytes, iPSCs, and endothelial cells. Matrix Gel Selection: The next tier involves selecting the proper matrix materials to support cell growth and differentiation. Commonly used matrices include Matrigel, collagen, silk fibroin, PEG, and DNA hydrogels. Construction Techniques: Key techniques such as stepwise induction, 3D bioprinting, and the use of bioreactors are employed to guide the development of complex organoid structures. Cytokines and Inducers Selection: Specific cytokines and chemical inducers are critical for driving cell differentiation and tissue development within the organoids. At the apex, fully developed bone or cartilage organoids are formed, representing the culmination of coordinated cellular interactions, matrix scaffolding, construction techniques, and biochemical signals

The construction of bone and cartilage organoids involves selecting appropriate cells, matrix gels, cytokines, inducers, and construction techniques to replicate the complex architecture and functionality of these tissues effectively, which is fundamental in the effective construction of bone/cartilage organoids, offering promising avenues for regenerative medicine and tissue engineering advancements (Fig. 4).

### Cell selection

#### Stem cells

In bone and cartilage repair, the application of stem cells has emerged as a pivotal area of research. These cells, particularly embryonic and iPSCs, are endowed with exceptional plasticity and regenerative capacities, making them ideal candidates for the construction of bone and cartilage organoids. In the field of cartilage tissue engineering,^40^ leveraging the properties of stem cells such as iPSCs, mesenchymal stem cells (MSCs) derived specifically from bone marrow, periosteum, and growth plates, and hPDCs, allows for the simulation of the cellular composition and hierarchical structure inherent to natural cartilage tissues. It is important to note that most adult stem cells are tissue-specific and form a limited number of phenotypes within their tissue of origin. Only bone marrow-derived MSCs, periosteal and growth plate skeletal stem cells, and certain umbilical cord blood cells have the inherent ability to generate bona fide cartilage. MSCs from other tissues typically require significant molecular engineering or the addition of specific factors to exhibit chondrogenic or osteogenic capabilities.

Particularly, iPSCs, with their robust abilities in proliferation, regeneration, and differentiation, play a crucial role in the construction of cartilage organoids.^41^ They offer a highly scalable and genetically precise source of pluripotent cells for organoid construction, further capable of delineating patient-specific disease models. This feature positions them as precise tools for drug screening. Moreover, MSCs, with their multipotentiality to differentiate in multiple directions, are also pivotal in cartilage tissue engineering.^42^ Post-cultivation, these cells can be induced through physical cohesion and cartilage-specific molecular signaling to produce organoids with specific extracellular matrices characteristic of cartilage. In the sphere of bone tissue engineering, the multifunctionality of stem cells is extensively harnessed. For instance, iPSCs can be reprogrammed into various cell types, constructing complex systems while maintaining consistent genotypes.^43^ This capability effectively models patient-specific bone disorders, facilitating drug testing and discovery. Similarly, MSCs in the natural bone microenvironment can differentiate into osteoblasts, offering a convenient route to bone organoid construction. Studies indicate that the stemness, proliferation, and differentiation capabilities of MSCs are significantly enhanced under three-dimensional culture conditions.^44^

Leveraging these unique properties of stem cells enables the more effective construction of organoid models that closely mimic natural bone and cartilage tissues. These advancements hold substantial significance in scientific research, offering novel approaches and methodologies for clinical applications, particularly in the realms of bone and cartilage repair. The translation of these insights into practical applications marks a revolutionary stride in regenerative medicine and tissue engineering, underscoring the transformative potential of stem cell research in these domains.

#### Effector cells

In the sphere of bone and cartilage repair, apart from stem cells, other cellular components play a crucial role in the regeneration and reconstruction processes. These non-stem cell types, each with their specialized functions, contribute significantly to the intricate balance and functionality of bone and cartilage tissue engineering. For instance, in the construction of bone organoids, different bone-related cells, namely osteoblasts, osteoclasts, osteocytes, chondrocytes, and endothelial cells are integral. Osteoblasts, responsible for building bone tissue, are primarily considered for bone disease research organoids.^45^ They are pivotal in laying down the bone matrix and initiating mineralization. Osteoclasts, derived from hematopoietic cells like macrophages, serve as the main executors of bone resorption, a process vital for maintaining bone health and architecture. They help in remodeling and adapting bone to mechanical stress by resorbing bone tissue. Osteocytes, the most abundant cell type in bone, regulate both the formation and resorption processes, acting as orchestrators in the bone microenvironment.^46^ Chondrocytes are essential for preserving the structure and functionality of cartilage, largely through their role in regulating the turnover of the ECM. These cells are key to distributing mechanical loads across joints and contribute to cartilage regeneration by synthesizing crucial matrix components such as collagen type II and aggrecan.^47^ Endothelial cells also play a critical role in bone and cartilage repair by facilitating vascularization. They are vital to bone tissue remodeling as they promote angiogenesis, ensuring the delivery of oxygen and nutrients to regenerating tissues. Additionally, endothelial cells help coordinate the activity of osteoblasts and osteoclasts, maintaining the balance between bone formation and resorption. Recent research has demonstrated that endothelial cell-conditioned media can promote the differentiation of chondrocytes into osteocytes, underscoring their importance in endochondral ossification during bone repair.^48–50^

In summary, the orchestration of various cell types plays a pivotal role in the regeneration and reconstruction of bone and cartilage tissues. Their collective contribution, governed by complex signaling networks, is essential for the successful engineering of functional bone/cartilage organoids, advancing the frontiers of regenerative medicine and tissue engineering.

### Matrix gel selection

#### Natural hydrogels

**Fig. 5:**
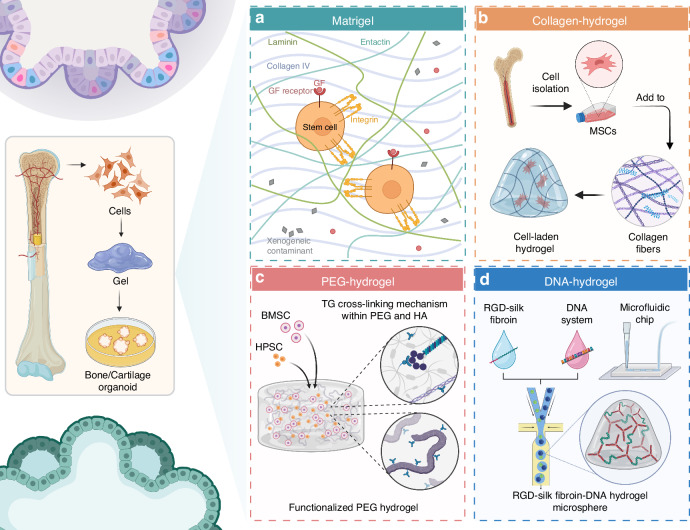
Overview of hydrogel systems used in the construction of bone/cartilage organoids. The figure illustrates four distinct hydrogel systems-Matrigel, Collagen-hydrogel, PEG-hydrogel, and DNA-hydrogel-used for supporting the development of bone and cartilage organoids. **a** Matrigel contains key extracellular matrix components such as laminin, collagen IV, and entactin, facilitating stem cell adhesion and proliferation through integrin and growth factor receptor interactions.^51^ **b** Collagen-hydrogel involves the incorporation of isolated mesenchymal stem cells (MSCs) into collagen fibers, forming a scaffold that promotes cell differentiation and tissue development.^53^ **c** PEG-hydrogel features a transglutaminase (TG) cross-linking mechanism using PEG and hyaluronic acid (HA) to form a functionalized hydrogel environment that supports the differentiation of bone marrow stromal cells (BMSCs) and hematopoietic progenitor cells (HPSCs).^56^ **d** DNA-hydrogel combines RGD-silk fibroin and a DNA system to create a microsphere hydrogel structure, which can be integrated into a microfluidic chip for precise tissue engineering applications^60^

The use of natural hydrogels in bone and cartilage organoid development represents a significant stride in tissue engineering. Matrigel, although extensively utilized due to its rich composition of basement membrane proteins, is not suitable for clinical applications. However, understanding how cells respond to Matrigel in forming organoids may provide useful insights into cell behavior and tissue development, which can guide the development of more clinically relevant scaffolds. While Matrigel is particularly valued for its capacity to mimic the ECM, this mimicry is limited to basement membrane structures, making it critical to explore alternative materials for broader organoid applications. When used in the culture of osteochondral organoids, Matrigel supports the differentiation of stem cells into osteogenic and chondrogenic lineages, thus aiding in the replication of the complex structure and function of bone and cartilage tissues. This is achieved through its composition of laminin, collagen IV, heparan sulfate proteoglycans, and growth factors, which collectively enhance cell-matrix interactions and promote tissue development^51^ (Fig. 5a). Tam et al. demonstrate the potential of cartilaginous organoids derived from hPSCs to heal critical-sized bone defects in a scaffold-free manner, which is a crucial step towards developing effective treatments for bone injuries and disorders.^52^ The use of Matrigel-coated well plates for the culture and development of cartilaginous organoids from hPSCs. This approach leverages the unique properties of Matrigel to support the growth and differentiation of stem cells into cartilaginous tissue. Despite its widespread use in research, Matrigel presents significant limitations for clinical applications, particularly due to its origin from mouse tumors. This source introduces variability and potential immunogenicity, making it unsuitable for GMP-compliant processes and in vivo transplantation in humans, who are not immunocompromised like the rodent models often used in preclinical studies. The future of bone/cartilage organoid applications lies in the advancement and adoption of these alternative scaffolding materials that align with clinical and regulatory requirements.

Collagen-based hydrogels, due to their biocompatibility and inherent ability to promote cell adhesion and proliferation, have been improved through various crosslinking techniques^53^ (Fig. 5b). These modifications aim to better replicate the mechanical properties of target tissues, as evidenced in the work of researchers who have developed collagen composite formulations to enhance tissue-specific responses. Kleuskens et al. investigate the potential of collagen-based hydrogels in facilitating the formation of neo-cartilage using cartilage organoids derived from both nondegenerate (ND) and osteoarthritic (OA) chondrocytes. While the study provides valuable insights into the application of hydrogels for cartilage regeneration, it could benefit from a more detailed characterization of the bone and cartilage tissues formed. Furthermore, the research does not thoroughly address the critical issue of hypertrophy within the cartilage, which is a significant factor in determining the long-term stability and functionality of the engineered tissue.^54^ This research found that cartilage organoids of both ND and OA origin, when embedded in viscoelastic hydrogels, were able to promote the formation of continuous tissues containing cells, proteoglycans, Col-II, and Col-I, which are essential for maintaining the integrity and function of cartilage. In particular, OA chondrocytes showed similar proliferation rates and tissue formation capacity to ND chondrocytes. At the level of gene expression, the COL2A1/COL1A1 ratio was higher in the OA group, indicating that OA chondrocytes have more potential in forming cartilage matrix. This finding is significant as it demonstrates the versatility of collagen-based hydrogels in supporting cartilage regeneration, regardless of the source of the chondrocytes, which could have implications for treating a range of cartilaginous pathologies, including osteoarthritis. The study highlights the importance of the ECM, particularly collagen, in providing the necessary cues for cell differentiation and tissue development. Collagen’s biocompatibility, biodegradability, and ability to mimic the natural ECM make it an ideal candidate for creating scaffolds that can support the growth and development of organoids into functional cartilage tissue.

#### Synthetic hydrogels

Polyethylene glycol (PEG), recognized for its non-irritating, hydrophilic, and bioinert characteristics, has emerged as a viable alternative to Matrigel references.^55^ Its chemical structure allows for straightforward modifications with active functional groups such as ester and unsaturated bonds, enhancing its utility in the field of tissue engineering. Consequently, PEG has gained popularity as a preferred biomaterial over Matrigel^56^ (Fig. 5c). In an innovative study, Vallmajo-Martin et al.^57^ incorporated a transglutaminase (TG) cross-linking mechanism within PEG and hyaluronic acid (HA) based hydrogels to engineer bone marrow organoids. The synergy between PEG and HA in these hybrid hydrogels led to improved physical and biochemical properties, crucial for the development of bone marrow organoids. While the study utilized BMP, it leaves open the question of whether bone formation was driven by BMP-induced endogenous fibroblasts or by BMSCs within the hydrogel, or potentially both. The research highlighted the effectiveness of TG-PEG/HA hydrogels in supporting the structural integrity, growth, and cell differentiation of BMSCs and hematopoietic progenitor stem cells (HPSCs) in vitro. Furthermore, compared to traditional biomaterials, these hybrid hydrogels demonstrated enhanced performance in xenograft models. Jansen and his team^58^ devised a method to embed bone marrow components and specific protein signatures within PEG hydrogels, crafting a mimicry of marrow’s natural elasticity enriched with 20 bone marrow-specific, cell-directing peptides.

DNA hydrogels stand out for their biocompatible and programmable properties.^59^ A recent study explores an innovative approach to enhancing cartilage regeneration through the development of a novel RGD-silk fibroin-DNA hydrogel microsphere (RSD-MS)^60^ (Fig. 5d). These microspheres are carefully designed through photopolymerization and self-assembly techniques, which not only provide excellent biocompatibility, ideal swelling, and degradation properties, but also serve as injectable solid scaffolds, which are particularly critical for the engineered construction of cartilage and bone tissue. In vitro experiments revealed that RSD-MSs significantly promoted the proliferation, adhesion, and chondrogenic differentiation of BMSCs. Transcriptomic analysis further elucidated that RSD-MSs primarily induced chondrogenesis through integrin-mediated adhesion pathways and glycosaminoglycan biosynthesis. Moreover, in vivo studies highlighted that seeding BMSCs onto RSD-MSs to form cartilage organoid precursors markedly enhanced cartilage regeneration, proving the potential of RSD-MSs as an injectable solid scaffold in cartilage regeneration. RSD-MS has emerged as a promising material for the fabrication of cartilage organoids, offering innovative approaches and material choices for advancements in the fields of cartilage regeneration and tissue engineering.

In summary, natural hydrogels, like Matrigel and collagen, are pivotal in bone and cartilage organoid development due to their cell-friendly composition and ability to mimic the ECM, promoting cell growth and tissue regeneration. Meanwhile, synthetic hydrogels such as PEG and DNA hydrogels are gaining prominence for their tunability and biocompatibility, proving instrumental in engineering bone marrow organoids and enhancing cartilage regeneration through innovative crosslinking and self-assembly techniques (Fig. 5). However, beyond soft scaffolds like hydrogels, hard scaffolds such as ceramics are increasingly recognized for their significant potential in bone/cartilage organoid construction. These ceramic materials, known for their mechanical strength and osteoconductive properties, are especially useful in replicating the mineralized matrix of bone, offering a stable framework that supports cellular attachment and proliferation. Ceramics like HYP and β-tricalcium phosphate are frequently employed both in experimental and clinical settings for their ability to enhance bone formation and support vascularization, critical aspects of effective organoid development. The field of hydrogel development for bone/cartilage organoid construction is characterized by constant innovation, aiming to create materials that not only support cell growth and differentiation but also accurately mimic the mechanical and biochemical cues of the native bone/cartilage tissue environment. The integration of both soft and hard scaffolds, leveraging the strengths of each, along with findings from various studies,^61^ is pivotal in advancing the capabilities of these hydrogels in regenerative medicine and effective tissue engineering strategies.

### Cytokines and inducers selection

Cytokines and inducers are pivotal in regulating cellular behavior, promoting differentiation, and facilitating tissue formation. By binding to cell surface receptors, they activate signaling pathways that guide cell fate and development. Incorporating these factors into matrix gels is essential for both the initial formation and long-term stability of organoids.^62^

In bone/cartilage organoid construction, cytokines like BMPs, TGF-β, FGFs, and IGFs are crucial for cell proliferation and tissue formation (Table 1). BMPs, for example, stimulate osteoblast differentiation and promote bone formation, while TGF-β regulates cell proliferation, differentiation, and ECM synthesis, aiding in tissue regeneration.^63^ Inducers such as β-mercaptoethanol, β-glycerophosphate, and dexamethasone also play significant roles. Dexamethasone enhances BMP-2-induced osteoblast differentiation and bone regeneration, and β-glycerophosphate, in combination with dexamethasone and ascorbic acid, significantly improves bone quality, as shown in studies on extraction socket healing in rabbits.^64^ This synergy between different inducers highlights their importance in optimizing the environment for bone and cartilage regeneration.

**Table 1:** Cytokines and inducers in bone/cartilage organoids

Organoid type	Source	Cytokines	Scaffolds	Inducers	Ways	References
Human cartilaginous organoids	Human iPSCs or ESCs or rat BMSCs	FGF-2, TGF-β1, FGF-2, BMP-2, GDF-5, TGF-β3	Decellularized extracellular matrix scaffolds	CHIR99021, β-Mercaptoethanol, Verteporfin	In vitro	^52,111^
Murine osteochondral organoids	Murine iPSC	TGF-β3, BMP-2	/	β-mercaptoethanol, β-glycerophosphate, dexamethasone	In vitro	^29^
Bone spheroids	Osteoblasts	TGF-β1, ITS	/	/	In vitro	^112^
Woven bone organoids	Human BMSCs	/	Silk fibroin	dexamethasone, and β-glycerophosphate	In vitro	^27^
Trabecular bone organoids	Human osteoblasts and osteoclasts	VD3, PGE-2, RANKL, M-CSF	/	β-glycerophosphate	In vitro	^5,72^
Bone marrow organoids	Human BMSCs or human CB-BFs	TGF-β1, TGF-β3, BMP-4, FGF-2, VEGFA, SCF, FLT3 ligand, IL-3, IL-6, G-CSF, EPO, TPO, PDGF-AB, OSM, IGF-1	/	2-phosphate-ascorbic acid, dexamethasone	In vitro	^65,69,75,113,114^
Bone organoids	Osteoblasts, osteoclasts, endothelial cells, dental pulp stem cells	M-CSF, RANKL, rhVEGF	/	dexamethasone, β-glycerophosphate disodium salt hydrate, ascorbic acid, calcium chloride	In vitro	^76,115^
Callus organoid	PDCs	BMP-2, GDF-5, TGF- β1, BMP-6, FGF-2	/	ascorbate-2 phosphate, dexamethasone, proline, Y27632	In vitro	^30^
Human skeletal organoids	MSCs and endothelial cells	M-CSF, RANKL, TGF-β1, ITS, BMP-2	/	dexamethasone, ascorbate-2-phosphate, β-glycerophosphate, A2AR agonist, A2AR antagonist	In vitro	^71^

Additionally, precise control over the concentration and release timing of these cytokines can simulate the natural cellular microenvironment, efficiently constructing bone/cartilage tissues with natural tissue structure and function in vitro. A recent study develops human bone marrow organoids as a model for studying hematologic malignancies and testing therapeutic targets.^65^ Throughout the differentiation and maturation phases of organoid development, various growth factors and cytokines are used, including BMP-4, FGF-2, VEGFA, SCF (Stem Cell Factor), FLT3 ligand, IL-3, IL-6, G-CSF (Granulocyte Colony-Stimulating Factor), EPO (Erythropoietin), and TPO (Thrombopoietin). These factors are essential for directing the differentiation of iPSCs towards the desired lineages and for supporting the growth and maturation of hematopoietic cells within the organoids.

Therefore, the application of cytokines in the construction of bone/cartilage organoids not only enhances the quality and functionality of tissue-engineered constructs but also contributes to a deeper understanding of the biological processes of bone and cartilage tissues. This provides new strategies and methods for the treatment of bone and cartilage-related diseases.

### Construction techniques selection

The development of bone and cartilage organoids necessitates the selection of appropriate construction techniques, each playing a distinct role in replicating the complex architecture and functionality of these tissues.

#### Stepwise induction

Stepwise induction is crucial in constructing bone and cartilage organoids due to its ability to mimic the natural developmental processes of these tissues. This method involves the sequential application of specific cytokines and growth factors that guide stem cells through different differentiation stages, closely resembling the in vivo environment. Firstly, stepwise induction ensures that stem cells progress through precise developmental stages. For example, mesodermal induction followed by chondrogenic differentiation in cartilage organoid formation uses factors like FGF, EGF, WNT-3a, collagen, and TGF-β to replicate the natural progression from mesoderm to mature cartilage.^66,67^ This control is essential for achieving the correct cell types and tissue structures necessary for functional organoids. In addition, by replicating the natural sequence of tissue development, stepwise induction produces organoids with properties similar to native bone and cartilage. This includes appropriate matrix composition, cellular organization, and functional capabilities such as load-bearing in bone and flexibility in cartilage. This similarity is vital for the organoids to be used effectively in disease modeling, drug testing, and regenerative medicine.

#### Bioprinting

Bioprinting has emerged as a revolutionary technique in tissue engineering, offering precise control over the spatial distribution of cells and biomaterials. This technology enables the fabrication of complex, three-dimensional structures that closely mimic the natural organization of bone and cartilage tissues.^68^ By depositing layers of bioinks—a mixture of cells, growth factors, and hydrogels—bioprinting allows for the construction of organoids with specific geometries and compositions. Although solid scaffold particles are not usually included in bio-inks, the potential role of nanoparticles cannot be ignored. These nanoparticles can be included in bio-inks, giving bioprinting an extra dimension to enhance its potential for applications in building complex tissue structures. Thus, this technique is not only particularly advantageous in creating heterogeneous structures that replicate the intricate architecture of bone and cartilage tissues, but also further optimize the creation of heterogeneous structures through the integration of nanoparticles, which is particularly important for replicating complex tissue structures such as bone and cartilage.

#### Bioreactors

Bioreactors play a crucial role in the construction of bone and cartilage organoids due to their ability to provide a controlled and physiologically relevant environment that supports cell growth, differentiation, and maturation. Bioreactors create a controlled environment that can precisely regulate temperature, pH, oxygen levels, and nutrient supply. These conditions are critical for the proper development and maintenance of organoids, ensuring that they mimic in vivo conditions as closely as possible. For bone and cartilage tissues, which require specific mechanical and biochemical cues to develop correctly, this control is vital. Furthermore, the use of bioreactors allows for the scale-up of organoid production, which is crucial for translational applications in drug testing and regenerative medicine. However, while bioreactors are amenable to scaling up the number of organoids, they are less effective when it comes to increasing the size of individual organoids, a limitation that should be considered. Bioreactors can maintain large volumes of culture medium and support the growth of multiple organoids simultaneously, making the process more efficient and reproducible. In addition, bioreactors enable dynamic culture conditions, such as periodic changes in nutrient supply or mechanical loading, which can mimic the natural physiological environment more closely than static cultures. This dynamic environment can improve the maturation and functionality of bone and cartilage organoids, making them more suitable for clinical applications. Thus, the use of bioreactors allows for the production of high-quality, reproducible organoids that can mimic the native tissue environment more closely.

## Research progress of bone/cartilage organoids

In the rapidly evolving fields of biomedical engineering and regenerative medicine, research on bone and cartilage organoids is advancing significantly. These studies are crucial for understanding the biological fundamentals and developing new therapeutic strategies for fractures, osteoporosis, and cartilage injuries. This review provides a comprehensive overview of recent advancements, focusing on how these findings emulate physiological processes and their potential in disease modeling, drug testing, and regenerative therapies. It also analyzes various cell sources such as fibroblasts, MSCs, and iPSCs in organoid models. Additionally, the review addresses challenges in clinical translation and explores future research directions, including biomaterials, 3D bioprinting advancements, and enhancing biomimicry of organoid models (Table 2).

**Table 2:** Research progress of bone/cartilage organoids

Organoid source	Experimental model	Research aim	Key findings	Clinical implications	Ref.
Bone organoids					
BMSC and CB-BF	In vivo bone/marrow organoids	Investigate CB-BF precursors in HSC niche formation	CB-BFs form functional hematopoietic microenvironments, and support human HSCs	Potential in regenerative medicine, particularly for hematopoietic tissue support	^69^
Human bone and cartilage tissues	Self-assembling skeletal organoids	Model human bone and cartilage tissues in vitro	Organoids replicate cellular environment of native tissues, useful for disease modeling	Advancement in biomedical engineering, aiding skeletal condition studies and therapy evaluation	^71^
Primary female osteoblastic and osteoclastic cells	Micron-scale bone organoids	Study bone processes at cell-tissue interface	Insights into pathological bone loss and bone remodeling	Model for studying bone diseases and testing therapeutics, understanding microgravity effects	^72^
Mesenchymal stem cells, endothelial cells	Trabecular bone organoid using DBP	Replicate trabecular bone remodeling processes	Demonstrated osteoblast to bone lining cell transition, regulation of bone remodeling	Advances understanding of trabecular bone physiology, limitations in replicating bone-marrow interface	^28^
Human mesenchymal stem cells-laden graphene oxide	3D bioprinted bone organoids	Develop functional osteocyte bone organoids under mechanical loading	Enhanced mineral density, osteoblast differentiation, and osteocyte formation	Potential in bone tissue engineering, understanding effects of mechanical loading	^74^
Human iPSC-derived cells	Human BM-like organoids	Model blood and BM disorders	Architectural and transcriptional homology to human BM, effective in disease modeling	Transformative for hematopoiesis and BM disorder studies, therapeutic target discovery	^65^
MSCs and ECs	3D BM organoids	Mimic native BM in vitro	Successful replication of BM features, supports HSPC recruitment	Novel platform for investigating stem cell behavior and leukemia treatment	^75^
BMSCs and DPSCs	Vascularized bone organoids	Overcome challenges in scaffold-free bone organoids	Successful vascularization and functionality in organoids	Advances bone regenerative medicine and drug development	^76^
Cartilage Organoids					
Human periosteum-derived cells, iPSCs	Hierarchical cartilage construct	Deep osteochondral defects repair	Creation of patterned constructs with distinct cartilaginous zones	Potential for complex tissue-engineered implants in joint repair	^77^
Bovine chondrocytes	Self-assembling cartilage organoids	Cartilage regeneration	Mass-production of organoids mimicking hyaline cartilage	Advancements in large-scale cartilage regeneration for osteoarthritis treatment	^78^
hiPSC-derived cartilage organoids	hiPSC with OA mutation model	Interaction of genetic and mechanical factors in OA	Mutation in COL6A3 affects collagen and fibronectin binding, influencing OA progression	Reconsideration of COX-2 inhibitors for OA pain treatment	^116^
Human chondrocytes (OA and ND)	Suspension expansion in NCM	Regenerative cartilage therapies	Comparable performance of OA and ND chondrocytes in forming cartilage organoids	Opens avenues for cartilage regeneration using OA chondrocytes	^54^
iPSC-derived cartilage organoids	Allogeneic transplantation in primates	Articular cartilage defects treatment	Successful integration and remodeling as articular cartilage in primates	Viability of allogeneic iPSC-derived cartilage organoids for treating chondral defects	^79^
Synovial mesenchymal stromal cells (SMSCs)	3D cultured organoids	OA treatment in regenerative medicine	Formation of proteoglycan-rich ECM; role of miR-138 in OA	Potential of SMSC-organoids in cartilage regeneration and OA therapy	^80^
iPSC-derived chondrocytes	Bone formation models	Healing large bone defects	iPSCs differentiate into chondrocytes, forming stable cartilage in vivo	iPSC technology for innovative bone healing strategies, overcoming limitations of traditional grafts	^52^
Osteochondral organoid					
Murine iPSCs	Scaffold-free osteochondral organoid system	Regeneration of integrated articular cartilage and bone tissues	Successful sequential differentiation into chondrogenic and osteogenic lineages; resistance to dedifferentiation	Offers platform for joint disease drug screening and genetic risk investigation	^29^
MSCs with gelatin-based microcryogels	Gelatin-based microcryogels modified with HA and HYP	Regeneration of hierarchical osteochondral units	Self-assembly into osteochondral organoids; promotion of differentiation and suppression of immune response	Injectable solution for regeneration of complex interfacial tissue	^32^
Microtissues within a 3D-printed framework	Biofabrication of osteochondral organoids	Articular cartilage (AC) repair and biofabrication	Creation of distinct microtissues supporting AC or bone regeneration; enhanced growth and fusion capacity	Advancement in biological joint resurfacing and treatment of osteochondral defects	^81^
Callus Organoids					
MSC aggregates, BMSCs	DLP printing technology, hydrogel microspheres	Rapid bone regeneration	Higher chondrogenic efficiency, rapid bone regeneration in large defects	Advances in bone regeneration for large defects, potential clinical applications	^83^
Human periosteum-derived cells (hPDCs)	Serum-free, xeno- and lipid-free medium	Tissue engineering, overcoming reliance on animal-derived components	Smaller, more homogeneous microtissues in xfCM, enhanced osteogenic differentiation	Scalability and automation in tissue-engineered product manufacturing	^84^
Human periosteum-derived cells	Microspheroids differentiating into callus organoids	Healing large bone defects	Formation of bone micro-organs, successful healing in a murine bone defect model	Promising approach for clinical applications in bone regeneration	^30^

### Bone organoids

The challenge of repairing bone defects, particularly in cases of complex fractures or diseases like osteoporosis, has long been a focal point in clinical orthopedics and regenerative medicine. Recent advancements in engineering bone organoids have opened up new avenues for addressing these challenges, offering innovative solutions to replicate the complex processes of bone healing and regeneration.

Research involving human umbilical cord blood-blood-borne fibroblasts (CB-BFs)^69^ has shed light on their unique potential as skeletal progenitor cells. The creation of bone/bone marrow organoids from these cells marks a significant leap in regenerative medicine, especially for conditions requiring HSC niche reconstruction.^70^ Simultaneously, the development of self-assembling skeletal organoids^71^ signifies a major advancement in accurately modeling human bone and cartilage tissues. These organoids provide a more realistic preclinical model for tissue development and disease research, offering a promising platform for drug screening and therapeutic development. In parallel, the creation of a micro-scale bone organoid model^72^ has provided invaluable insights into bone loss processes and remodeling dynamics. This approach addresses the challenges of studying diseases like osteopenia and osteoporosis, providing a tangible model to understand and treat these conditions more effectively. Additionally, in a recent study by Jiacan et al., large-scale self-mineralizing bone organoids were constructed using GelMA/AlgMA/HAP composite bioink through three-dimensional bioprinting technology. These bone organoids exhibited excellent cell viability, self-mineralization capacity, and multicellular differentiation potential in both in vitro and in vivo cultures.^73^

The trabecular bone organoid model^28^ using demineralized bone paper (DBP), a biocompatible scaffold derived from natural bone that retains its collagenous matrix after mineral removal, further exemplifies the innovative approaches being employed to replicate the complexity of bone tissue. By mimicking the trabecular bone’s microenvironment, this model offers profound insights into the localized bone remodeling activities, crucial for understanding various bone diseases and their treatment. The exploration of mechanical loading in bone organoid development^74^ underscores the significant role of biomechanical factors in bone health. This research highlights the necessity of integrating mechanical cues into organoid development to enhance their functionality and resemblance to native bone tissue. Advancements in bone marrow organoids^65,75^ have revolutionized our approach to studying hematopoiesis and blood disorders. These organoids, replicating the intricate environment of bone marrow, offer a novel platform for investigating stem cell behavior, potential leukemia treatments, and drug discovery. In particular, by integrating dental pulp stem cells (DPSCs) with BMSCs, researchers have been able to create vascularized bone organoids, an important step forward in addressing key challenges of cell survival and function in organoid structures.^76^ Although DPSCs are similar to bone formation processes in terms of dentin formation, they are not identical, and the specific role of these cells in bone regeneration and the rigor of the formed vascular structures still need to be further studied. The data demonstrate the potential of this process, but further studies are needed to fully understand the angiogenic role of DPSCs in tissue engineering. This innovative study highlights the importance of vascularization in bone regenerative medicine and opens up new avenues for future research and clinical applications.

In conclusion, research on bone organoids represents a significant shift in regenerative medicine, addressing challenges in bone defect repair, hematopoietic niche reconstruction, and vascularization. Studies highlight the use of CB-BFs, DPSCs, bioprinting technologies, and mechanical loading, each contributing to bone healing and marrow functionality. Future directions include enhancing physiological relevance, refining 3D bioprinting, and integrating patient-specific cells for personalized therapies.

### Cartilage organoids

Cartilage defects, particularly in the context of osteochondral injuries, present a significant clinical challenge due to the limited regenerative capacity of articular cartilage and the complexities involved in simultaneous bone and cartilage repair. Addressing these defects is crucial not only for restoring joint function but also for preventing the progression of diseases such as osteoarthritis. Recent advancements in tissue engineering and regenerative medicine, particularly through the development of cartilage organoids, have shown promising strategies to tackle these challenges.

A study^77^ proposes an innovative tissue engineering strategy that uses cartilage organoids derived from human periosteal cells as building blocks to create patterned structures with well-defined biological functional divisions. These organoids not only represent different stages of chondrodevelopment, but are also capable of forming hybrid tissues with mineralized and non-mineralized regions after implantation in vivo. This finding has important implications for understanding the interfacial interactions of cartilage and bone tissue and provides new ideas for designing complex implants with region-specific functions. The modular approach of the study not only demonstrates its potential in addressing the need for integrated osteochondral tissue repair, but also opens up possibilities for personalized medicine and patient-tailored treatments. Another research initiative^78^ explored new ways to mass-produce cartilage organoids from bovine chondrocytes. Through suspension culture techniques and the application of notochordal cell-derived matrix, the researchers succeeded in rapidly generating a large number of organoids highly similar to natural hyaline cartilage. These engineered organoids are similar to natural cartilage in the composition, structure, and biophysical properties of the ECM, providing a powerful platform for cartilage tissue engineering. In particular, this study highlights the importance of viscoelasticity of hydrogels in cartilage formation, revealing that by regulating the viscoelastic properties of hydrogels, organoids can be promoted to grow, fuse, and form homogeneous cartilage tissues rich in collagen type II and glycosaminoglycans. This finding has guiding significance for material selection and design of cartilage tissue engineering, and provides a new way to achieve large-scale cartilage regeneration on joint surface. Addressing the limitations of current regenerative therapies, another study^54^ introduces a novel protocol for self-assembling human chondrocytes into cartilage organoids. Utilizing notochordal cell-derived matrix, the organoids formed are comparable in both OA and ND chondrocytes, showing similar proliferation rates and matrix production. This approach opens new possibilities for cartilage regeneration and serves as a model for studying disease pathways and drug development.

Furthermore, the potential of iPSCs for allogeneic cartilage transplantation is investigated in a study.^79^ This research demonstrates that allogeneic iPSC-derived cartilage organoids can integrate and remodel as articular cartilage in a primate model, highlighting their viability for treating articular cartilage defects. In a study^80^ focusing on synovial mesenchymal stromal cells, bioengineered cartilage organoids exhibit significant chondrogenic marker expression and form a proteoglycan-rich ECM. The study emphasizes the role of miRNAs, particularly miR-138, in chondrogenic properties and OA development, offering insights into cartilage regeneration and OA therapy. Lastly, a study^52^ explores using iPSCs to heal large bone defects. The derived chondrocytes form stable cartilage in vivo and mediate the healing of critical size long bone defects, showcasing the potential of iPSC technology in bone healing strategies.

In conclusion, the exploration of cartilage organoids in these studies represents a transformative step in addressing the complexities of cartilage repair and osteoarthritis treatment. Each study contributes uniquely to the field, collectively advancing our understanding of the intricate biological processes involved in cartilage regeneration and the potential of various cell sources. The findings underscore the importance of creating biologically accurate and functional tissue models, from iPSC-derived organoids to those developed from synovial mesenchymal stromal cells, highlighting the diversity of approaches in tissue engineering. Looking forward, the field is poised for significant developments in personalized medicine and regenerative therapies. Future research should prioritize refining these organoid models to enhance their biological fidelity, scalability, and clinical translatability. Emphasis on understanding the molecular mechanisms underlying cartilage formation and degradation, as well as the integration of advanced biomaterials and 3D printing technologies, could further propel this field. Moreover, exploring the genetic and environmental factors in cartilage pathology through these organoid models could unlock new avenues for targeted therapies in osteoarthritis and other cartilage-related disorders. As we advance, the potential for these organoids to revolutionize cartilage repair and provide patient-specific treatment options becomes increasingly tangible.

### Osteochondral organoids

The integration of articular cartilage and bone tissues in osteochondral tissue engineering and osteoarthritis disease modeling is a significant clinical challenge. Achieving simultaneous structural and functional repair in these tissues is complex due to their distinct healing capacities, tissue integration properties, and the dynamic gradient changes along structural axes. This complexity has spurred research into developing osteochondral organoids, a promising avenue in biomedical engineering, aiming to replicate these integrated structures for effective therapeutic applications.

One study^29^ utilized murine iPSCs to develop a scaffold-free osteochondral organoid system. This system sequentially differentiated iPSCs into chondrogenic and osteogenic lineages, emulating the endochondral ossification process. The organoids comprised both a cartilaginous region and a calcified bony region, making them ideal for joint disease drug screening and genetic risk assessment. Despite a decrease in chondrogenic gene expression during the osteogenic phase, the organoids maintained higher expression levels than baseline iPSCs, indicating successful differentiation. The study’s findings are pivotal for iPSC-derived tissue applications, particularly in preventing dedifferentiation within chondrogenic tissues.

Another research^32^ developed gelatin-based microcryogels modified with HA and HYP, termed CH-Microcryogels and OS-Microcryogels, to induce cartilage and bone regeneration. These microcryogels demonstrated cytocompatibility and the ability to direct BMSC differentiation towards chondrogenic and osteogenic lineages. Moreover, they self-assembled into osteochondral organoids without delamination, showing promise for in situ regeneration of complex interfacial tissues like osteochondral units.

Burdis et al.^81^ presented a novel biofabrication approach using microtissues within a 3D printed framework to develop osteochondral organoids. This approach overcomes limitations in articular cartilage repair by strategically engineering distinct microtissues that support either articular cartilage or subchondral bone regeneration. The use of TGF-β enhanced the growth and fusion capacity of these microtissues, leading to the development of larger hyaline cartilage grafts. This method effectively generated unified and homogeneous cartilage macrotissues, displaying stable hyaline cartilage properties.

In conclusion, the advancement of osteochondral organoids represents a significant leap in biomedical engineering, particularly in addressing the intricate relationship between bone and cartilage in joint diseases. These studies aforementioned illustrate diverse approaches to mimic the natural structure and function of osteochondral units, ranging from scaffold-free systems using iPSCs to innovative microcryogel techniques and biofabrication within 3D frameworks. These advancements provide promising pathways for developing more effective treatments for osteoarthritis and other joint-related disorders. Looking ahead, the field of osteochondral organoid engineering faces the challenge of scaling these technologies for clinical use while maintaining the intricate balance between cartilage and bone tissue regeneration. Future research should focus on refining these organoid systems to enhance their biomimicry and functionality, ensuring that they can adapt to the dynamic environment of the human body. Additionally, the integration of advanced imaging and analytical techniques could provide deeper insights into the organoids’ development and functionality, facilitating their translation from bench to bedside. This progress in osteochondral organoid engineering has the potential to revolutionize the treatment of joint diseases and improve the quality of life for patients worldwide.

### Callus organoids

Callus organoids represent a novel frontier in biomedical engineering, aiming to replicate the intricate process of bone healing and regeneration. The callus, a pivotal phase in the natural repair mechanism of bones, involves complex cellular activities including proliferation, differentiation, and maturation. This process is critical for restoring bone structure and function following injury or disease.^82^ Advanced research in this field is not only essential for deepening our understanding of bone biology but also holds significant potential for developing effective therapeutic strategies for bone-related ailments.

In a recent study,^83^ researchers developed an innovative approach for rapid bone regeneration, particularly crucial in healing large bone defects which typically have extended recovery periods. The strategy involved creating osteo-callus organoids using BMSC aggregates. These organoids, formed in conjunction with BMSCs encapsulated in hydrogel microspheres (MSs) via digital light-processing (DLP) printing technology, exhibited superior chondrogenic efficiency compared to traditional MSC pellets. The gene expression of these organoids closely mirrored the endochondral ossification process, a natural bone formation mechanism. This innovative method significantly accelerated bone regeneration within four weeks in a rabbit model of large bone defects, marking a substantial advancement over existing bone regeneration techniques.

Another significant study^84^ sought to overcome the challenges in tissue engineering, particularly in manufacturing cell-based advanced therapy medicinal products. This research focused on developing a serum-free, chemically defined, xeno- and lipid-free chondrogenic differentiation medium to generate bone-forming callus organoids. hPDCs were differentiated into 3D microtissues using this new medium and compared with conventional chondrogenic media. The findings revealed that the new medium produced smaller, more uniform microtissues, suggesting a promising solution to avoid batch failures in large-scale tissue engineering. Both media types successfully generated in vivo ectopic bone ossicles, with the new medium showing enhanced osteogenic differentiation over time.

Nilsson et al.^30^ address the unpredictability of cell-based products in clinical applications. Utilizing a developmental engineering approach, this research produced tissue products mimicking natural bone regeneration processes. hPDCs were used to create microspheroids, which differentiated into callus organoids. These organoids autonomously formed bone micro-organs in vivo, showing potential for healing large bone defects. The organoids followed an early pattern of endochondral ossification, transitioning from cell aggregation to cartilage tissue intermediate formation, and eventually maturing into pre-hypertrophic callus organoids. This study also demonstrated the healing efficacy of these organoids in a murine model of critical-sized long bone defects, where they formed a native-like bone structure.

Collectively, these studies represent significant progress in the field of callus organoids, offering new insights and approaches to bone regeneration. However, challenges such as nutrient limitations, cell necrosis in large-sized defects, and scaling up production remain. Future research should focus on addressing these issues to fully exploit the clinical potential of callus organoids for bone healing and regeneration.

Overall, the construction of both bone and cartilage organoids relies on fundamental tissue engineering principles, including the selection of appropriate cells, matrix gels, cytokines, and advanced construction techniques such as bioprinting and bioreactors. These shared elements provide the foundational framework for developing complex three-dimensional structures that can mimic native tissues. Both organoids utilize iPSCs and ESCs and BMSCs due to their high plasticity and regenerative capabilities. Natural hydrogels like Matrigel and collagen are widely used for both types of organoids due to their biocompatibility and ability to mimic the natural ECM. Synthetic hydrogels, such as PEG-based hydrogels, are also suitable for both bone and cartilage organoids because of their customizable properties. Additionally, both organoids require cytokines and growth factors to promote differentiation and tissue-specific functionality, including BMPs, TGF-β, and FGFs, although their specific roles and combinations may vary.

Bone organoids have specific requirements, such as the use of BMSCsto promote bone formation and mineralization, osteoclasts to participate in bone resorption and remodeling, and osteocytes to regulate bone metabolism. Bone organoids often use matrix gels containing HA to enhance mechanical properties and support osteogenesis. Key cytokines and inducers such as BMP-2 (crucial for osteoblast differentiation), dexamethasone (enhances osteogenic differentiation when used with BMP-2), and β-glycerophosphate (supports mineralization processes) are particularly important in the construction of bone organoids. Advanced bioprinting techniques are used to create complex bone structures with precise spatial organization, while bioreactors simulate mechanical loading to promote bone tissue development. In contrast, cartilage organoids primarily use chondrocytes and MSCs that can differentiate into chondrocytes under specific conditions. Cartilage organoids commonly use collagen-based hydrogels and chondroitin sulfate to provide an ideal environment for chondrogenesis. Key cytokines and inducers such as TGF-β1 (promotes chondrogenic differentiation), SOX9 (a critical transcription factor for chondrogenesis), and CHIR99021 (a GSK3 inhibitor used to enhance cartilage formation) play significant roles in cartilage organoid construction. Construction techniques for cartilage organoids include scaffold-free methods, which encourage the self-assembly of chondrocytes into three-dimensional structures, and dynamic culture systems that provide mechanical stimuli to enhance cartilage tissue properties.

## Potential applications of bone/cartilage organoids

### Construction of disease model

Bone and cartilage organoids have emerged as innovative and promising tools in the field of regenerative medicine and disease modeling. These organoids, essentially self-organizing 3D cultures derived from stem cells, mimic the key features of bone and cartilage tissues.^85^ Their application in modeling various diseases is notable due to their high physiological relevance and potential to bridge the gaps in current understanding.

#### Osteoarthritis (OA)

Organoids offer a dynamic model for studying OA, a disease marked by cartilage degradation and joint dysfunction. By mimicking the OA environment, organoids enable the investigation of pathological processes like chondrocyte death, inflammation, and ECM breakdown. They also facilitate the testing of therapies and evaluation of early disease biomarkers.^80^ Furthermore, organoids provide a platform to study the effects of mechanical stress and cytokines on cartilage, aiding in understanding OA progression and developing targeted treatments. Their ability to replicate complex joint interactions makes them invaluable for advancing OA research and treatment strategies.

#### Bone fractures and healing

Bone fractures and their healing process involve complex biological and mechanical interactions. Creating accurate disease models is crucial for understanding these processes and developing effective treatments. Bone organoids simulate the complex physiological process of bone healing, which involves inflammation, new bone formation, and bone remodeling. They enable the study of cellular interactions between osteoblasts, osteoclasts, and other cell types involved in bone repair. Organoids are instrumental in understanding the roles of various growth factors and signaling pathways in bone regeneration. Moreover, they offer a testing ground for novel therapeutic approaches, such as bone morphogenetic proteins and scaffolds that promote bone healing, as well as for personalized medicine strategies to enhance fracture repair.^67^

#### Rheumatoid arthritis (RA)

RA is characterized by chronic joint inflammation and cartilage destruction. Organoids provide insights into RA’s pathogenesis by modeling synovial tissue interactions with immune cells, aiding the study of inflammatory mediators and genetic factors. They also optimize pharmacological agents targeting inflammatory pathways, offering potential for personalized medicine.^86^ RA is characterized by chronic joint inflammation and cartilage destruction. Organoids provide insights into RA’s pathogenesis by modeling synovial tissue interactions with immune cells, aiding the study of inflammatory mediators and genetic factors. They also optimize pharmacological agents targeting inflammatory pathways, offering potential for personalized medicine.

#### Genetic bone disorders

Organoids are used to model genetic bone diseases like osteogenesis imperfecta (OI), characterized by brittle bones. They enable the study of mutations in collagen-producing genes and their impact on bone structure and strength. Organoids provide insights into cellular mechanisms underlying these disorders and the efficacy of gene editing techniques like CRISPR/Cas9 for therapeutic interventions. They also help in evaluating the effectiveness of pharmacological treatments aimed at improving bone density and reducing fracture risk.^87^

#### Cartilage regeneration and repair

Organoids model cartilage injuries and degenerative conditions, aiding in the understanding of chondrocyte behavior and matrix regeneration. They are pivotal in evaluating tissue engineering strategies, such as scaffold-based and scaffold-free approaches, for cartilage repair. Organoids also enable the study of signaling pathways and molecular mechanisms involved in chondrogenesis and cartilage homeostasis. This is crucial for developing regenerative therapies and improving clinical outcomes in cartilage repair.^88^

### Drug prediction

The integration of bone/cartilage organoids into drug prediction heralds a transformative era in pharmaceutical research and development, offering a multitude of applications that promise to revolutionize how we approach drug efficacy, safety, and personalized treatment.^41^ These organoids, mirroring the complex microenvironment of bone and cartilage tissues, stand at the forefront of pharmacological innovation.

In the realm of high-throughput drug screening, the use of bone/cartilage organoids is particularly impactful.^89^ Unlike traditional 2D cell cultures, organoids provide a three-dimensional model that closely mimics the in vivo conditions of human tissues. This structural and functional similarity allows for a more accurate assessment of a drug’s therapeutic potential and toxicity. Such a platform is crucial for early-stage drug discovery, enabling researchers to screen a vast array of compounds efficiently and reducing reliance on animal models.

The implications of bone/cartilage organoids extend into the realm of personalized medicine.^45^ By deriving organoids from patient-specific cells, especially in diseases like osteoarthritis or RA where treatment responses are highly individualized, they offer a tailored approach to predict the efficacy and safety of drugs. This personalized testing not only increases the likelihood of treatment success but also significantly lowers the risk of adverse drug reactions, marking a substantial step towards patient-centric therapies.

Furthermore, organoids provide a window into the intricate mechanisms of drug action. Within their physiologically relevant environment, the detailed observation of cellular responses sheds light on the pathways and interactions through which drugs exert their effects. This insight is invaluable for the development of targeted therapies, particularly in addressing specific pathways involved in bone and cartilage pathologies.^90^

Safety pharmacology is another domain where bone/cartilage organoids are proving invaluable. At the cellular level, osteochondral organoids facilitate the co-culture of various cell types, including chondrocytes, osteoblasts, and immune cells. This co-culture system enables the study of cellular interactions and the identification of potential cytotoxic effects of drugs on different cell populations. It also allows for the monitoring of drug-induced changes in cell viability, proliferation, and differentiation, providing crucial information on cellular toxicity. Molecularly, osteochondral organoids offer insights into the molecular mechanisms underlying drug responses. Researchers can investigate the impact of drugs on gene expression profiles, signaling pathways, and matrix composition. This molecular analysis helps identify off-target effects and potential biomarkers for drug safety. Functionally, these organoids can be used to assess the biomechanical properties of joint tissues, such as stiffness and elasticity, which are critical parameters in safety pharmacology. Drugs that negatively impact the mechanical integrity of the joint tissues can be identified early, preventing potential adverse effects in clinical trials. By providing a more accurate and comprehensive model for drug testing, organoids could significantly improve the predictability of drug safety, reducing the risk of adverse effects in clinical trials and ultimately leading to safer and more effective therapies for patients.

In conclusion, bone/cartilage organoids represent a paradigm shift in drug prediction, offering a sophisticated, accurate, and versatile platform that resonates with the needs of modern drug development. Their ability to replicate human tissue environments more closely than conventional models enhances the precision and relevance of pharmacological studies, paving the way for innovative therapeutic solutions tailored to individual patient needs and specific disease characteristics.

### Evaluation of biomaterials

Bone/cartilage organoids offer a novel and highly effective approach for the evaluation of biomaterials, particularly in the context of tissue engineering and regenerative medicine. These organoids serve as an advanced platform to test and optimize the properties of biomaterials designed for bone and cartilage repair and regeneration.

In bone tissue engineering, the evaluation of biomaterials using bone organoids enables researchers to analyze how these materials support osteogenic differentiation and bone tissue formation. Mikael et al.^91^ contribute significantly by exploring patient-specific biomaterials that can support bone repair, emphasizing the importance of compatibility and effectiveness in promoting bone regeneration. Similarly, in cartilage repair, organoids provide a dynamic system to assess the mechanical and biological properties of biomaterials. For instance, the study performed Vainieri et al.^92^ highlighted the need for biomaterials to not only support chondrocyte growth but also respond appropriately to mechanical stimuli, which is crucial for the successful integration and function of repaired cartilage tissues.

These organoids are instrumental in replicating the complex microenvironment of bone and cartilage, thus allowing for a more accurate assessment of biomaterials’ performance.^93^ Researchers can evaluate factors such as biocompatibility, bioactivity, and the capacity of biomaterials to support cell attachment, proliferation, and differentiation. Moreover, they enable the study of biomaterials’ interactions with surrounding cells and their influence on the immune response, which is critical for predicting the in vivo behavior of these materials. Furthermore, bone/cartilage organoids are valuable for testing drug delivery systems incorporated into biomaterials. They offer insights into the controlled release of therapeutic agents and their impact on tissue regeneration and healing processes.

In conclusion, bone/cartilage organoids represent a significant advancement in the field of biomaterials research. They provide a more physiologically relevant model than traditional in vitro systems, thereby enhancing the accuracy and predictability of biomaterials evaluation. This is essential for developing innovative solutions for bone and cartilage repair, ultimately leading to improved clinical outcomes in regenerative medicine.

### Metabolomic analysis

Metabolomics, the comprehensive study of metabolites in biological systems, provides a deep understanding of cellular processes and responses. Bone/cartilage organoids, as in vitro models that closely mimic in vivo bone and cartilage tissues, present a unique opportunity for detailed metabolomic studies.

In the field of bone research, metabolomic analysis using bone organoids can uncover the complex biochemical pathways involved in bone metabolism, growth, and remodeling. In a significant study, researchers utilized metabolomic analysis to understand how bone cells respond to different biomaterials. This study demonstrates the application of metabolomics in assessing the compatibility and efficacy of biomaterials used in bone repair and regeneration, providing valuable information for the development of improved materials for bone tissue engineering.^94^

Moreover, bone/cartilage organoids can be used to investigate the metabolic pathways involved in cartilage development and pathologies. By analyzing the metabolomic profiles of cartilage organoids, researchers can gain insights into the metabolic alterations associated with cartilage diseases like osteoarthritis. This knowledge is crucial for developing targeted therapies that can modulate specific metabolic pathways to treat or prevent cartilage degradation. the integration of metabolomics with bone/cartilage organoid technology opens new avenues for research in bone and cartilage biology, disease understanding, biomaterials development, and pharmacology. These studies provide a foundation for future research aimed at harnessing the power of metabolomics in bone and cartilage research, ultimately leading to improved diagnostics, therapeutics, and regenerative strategies.

### Genomic engineering

Genomic engineering, particularly techniques like CRISPR/Cas9, has revolutionized the way we can manipulate and study genetic material in organoids. Bone/cartilage organoids serve as a powerful model system for genomic engineering due to their ability to closely mimic the in vivo microenvironment of bone and cartilage tissues.^95^

One key application of genomic engineering in bone/cartilage organoids is the study of genetic diseases affecting the skeletal system. For example, organoids can be engineered to carry specific mutations found in diseases like OI or chondrodysplasia. This allows researchers to study the disease mechanisms at a cellular level and identify potential therapeutic targets. In tissue engineering and regenerative medicine, genomic engineering can be used to enhance the osteogenic or chondrogenic potential of organoids. By manipulating genes involved in bone and cartilage formation, scientists can improve the efficiency of organoids in bone and cartilage regeneration. This has significant implications for treating conditions such as osteoporosis, arthritis, and bone fractures. Genomic engineering in organoids also facilitates drug screening and personalized medicine. Organoids with specific genetic alterations can be used to test the efficacy of drugs targeting those alterations.^89^ This approach is particularly valuable for developing treatments for genetic disorders affecting the skeletal system. Furthermore, genomic engineering can be used to create organoid models for cancer research. For instance, bone/cartilage organoids engineered to express oncogenes or knockout tumor suppressor genes can provide insights into the development and progression of bone and cartilage tumors.

### Biobanks

The potential applications of bone/cartilage organoids in biobanks represent a significant advancement in biomedical research and personalized medicine. Biobanks, which store biological samples for use in research and clinical applications, are increasingly incorporating organoids as a key resource. This integration offers numerous advantages due to the unique properties of organoids.^96^

Bone/cartilage organoids, derived from patient tissues, can be stored in biobanks and used for various research purposes. One of the primary applications is in the field of personalized medicine. Organoids provide a patient-specific model that can be used to test drug responses, allowing for the development of personalized treatment plan.^97^ For example, organoids derived from a patient with a specific bone or cartilage disease can be used to screen various drugs and determine the most effective treatment for that individual. Another critical application is in disease modeling. Organoids in biobanks can be used to create models of various bone and cartilage diseases, enabling researchers to study disease mechanisms and test new therapies. This is particularly valuable for rare diseases, where patient samples are limited. Organoids offer a way to study these diseases in vitro, providing insights that are difficult to obtain from traditional cell cultures or animal models. Organoids also play a significant role in regenerative medicine. Biobanks can store organoids derived from healthy tissues, which can be used for tissue engineering and regenerative therapies.^98^ For example, bone/cartilage organoids can be used to develop new treatments for conditions such as osteoarthritis or osteoporosis. Moreover, the diversity of organoids in biobanks reflects the genetic variability of the population, making them an excellent resource for genetic and epidemiological studies.^7^ Researchers can investigate the genetic factors contributing to bone and cartilage diseases, understand population health trends, and develop strategies for disease prevention and management.

Integration of bone/cartilage organoids into biobanks marks a significant leap forward in medical research. It bridges the gap between basic science and clinical applications, providing a versatile and dynamic platform for personalized medicine, disease modeling, drug development, regenerative therapies, and public health research. This integration not only enhances our understanding of bone and cartilage biology but also paves the way for novel therapeutic interventions, ultimately improving patient outcomes and advancing healthcare.

**Fig. 6:**
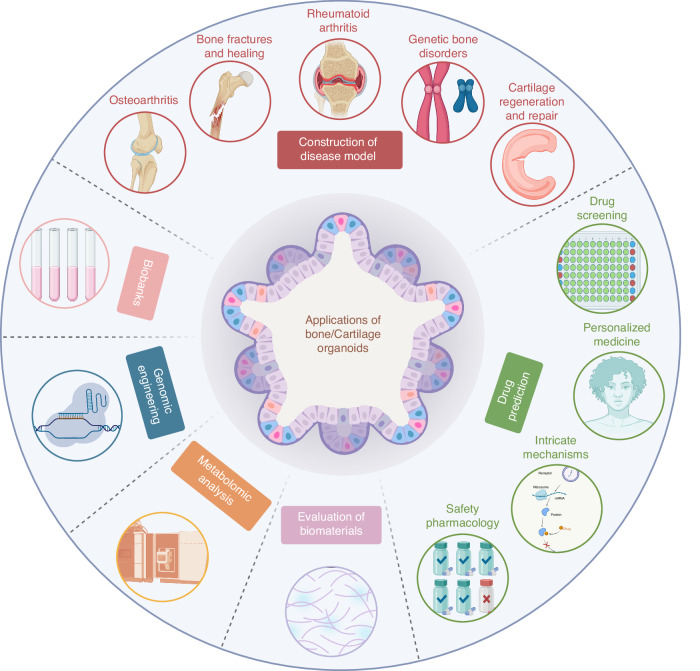
Potential applications of bone/cartilage organoids. The diagram showcases the wide-ranging applications of bone and cartilage organoids in biomedical research and therapeutic development. Construction of Disease Models: Organoids are used to model diseases such as osteoarthritis, bone fractures and healing, rheumatoid arthritis, genetic bone disorders, and cartilage regeneration and repair. These models offer insight into disease mechanisms and allow for testing therapeutic interventions. Drug Discovery and Personalized Medicine: Organoids enable high-throughput drug screening, drug prediction, and intricate studies of disease mechanisms. They are also pivotal in advancing personalized medicine by tailoring treatments based on individual responses. Biomaterial Evaluation and Safety Testing: Bone and cartilage organoids allow for the evaluation of biomaterials and safety pharmacology, providing a more physiologically relevant context for material and drug testing. Genomic and Metabolomic Analysis: These organoids facilitate the study of genomic alterations and metabolic pathways, offering deeper insights into cellular responses and disease states. Biobanks and Tissue Engineering: Bone and cartilage organoids can be stored in biobanks for future research and used in genomic engineering and metabolic analysis, paving the way for advanced tissue engineering applications

In summary, bone and cartilage organoids, are reshaping regenerative medicine and disease modeling with their close mimicry of human tissues (Fig. 6). They have proven essential in understanding and developing treatments for osteoarthritis by replicating key pathological processes and testing therapies. These organoids also simulate bone healing, offering insights into cellular dynamics and therapeutic strategies for fractures. In RA research, they model synovial tissue interactions, aiding in the discovery of targeted treatments. Additionally, organoids facilitate the study of genetic bone disorders like OI, allowing for the exploration of gene editing solutions. They are pivotal in cartilage regeneration, enhancing the development of tissue engineering approaches. Moving to drug prediction, organoids are revolutionizing pharmaceutical research by enabling high-throughput drug screening and personalized medicine, ensuring a more accurate assessment of drug efficacy and safety. In evaluating biomaterials, organoids serve as advanced platforms for testing materials aimed at bone and cartilage repair, crucial for regenerative medicine advancements. Metabolomic analysis in organoids offers deep insights into bone and cartilage metabolism, guiding the development of targeted therapies. Genomic engineering in organoids opens new avenues for studying genetic diseases and enhancing regenerative capacities. Lastly, the integration of organoids into biobanks represents a significant leap, supporting personalized medicine, disease modeling, and regenerative therapies, thus bridging the gap between basic science and clinical applications.

## Conclusion and perspective

In conclusion, the rapidly evolving field of bone/cartilage organoid engineering offers novel approaches to tissue regeneration, disease modeling, and drug discovery, providing more physiologically relevant platforms than traditional 2D cultures. However, significant challenges remain, particularly in replicating the intricate hierarchical structures and biomechanical properties of native tissues. For example, recreating the complex architecture of mineralized bone matrix and the dense ECM of cartilage in vitro is still challenging, leading to organoids that may not fully replicate native tissue functionality.^99^

Another challenge lies in the standardization of protocols for generating and maintaining organoids. Variations in stem cell sources, differentiation protocols, and culture conditions can result in inconsistencies in organoid formation and quality.^100^ Additionally, the long-term stability and functional viability of organoids remain problematic. Over time, organoids can experience issues such as necrosis in their core due to inadequate nutrient diffusion, which limits their utility for long-term studies.^101^

Efforts to address these challenges are ongoing and multifaceted. Advanced 3D bioprinting technologies are being developed to fabricate more accurate tissue models with precise spatial organization, which can better replicate the native tissue architecture.^102^ Furthermore, the integration of artificial intelligence (AI) and machine learning is being explored to optimize differentiation protocols and improve the reproducibility and scalability of organoid production.^103^ Researchers are also investigating the use of vascularization strategies, such as co-culturing with endothelial cells, to enhance nutrient diffusion and long-term viability of organoids.^104^

**Fig. 7:**
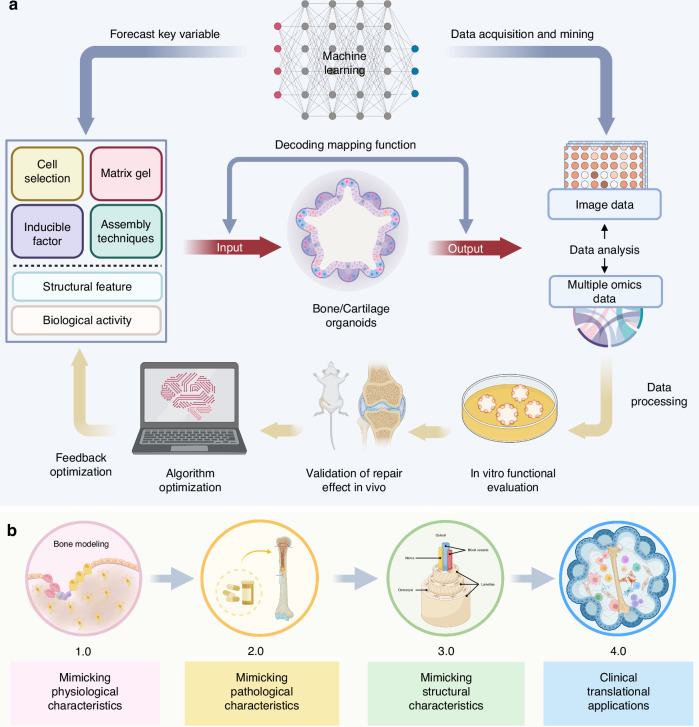
Integration of machine learning in bone/cartilage organoid development and evolution. **a** Machine Learning-Driven Optimization of Bone/Cartilage Organoids: This panel illustrates the use of machine learning to enhance organoid development. Key variables, including cell selection, matrix gel, assembly techniques, and biological activity, are forecasted and fed into a machine-learning model. The model decodes these inputs into outputs that optimize organoid structural and biological features. Data acquisition, such as image data and omics analysis, is processed to fine-tune the organoid development process through feedback optimization and in vivo validation. The algorithm continuously learns from this feedback loop, improving the design and function of organoids for regenerative purposes. **b** Evolution of Bone Organoid Modeling: This panel shows the progression of bone/cartilage organoid development through four stages: 1.0-Mimicking physiological characteristics of bone. 2.0-Mimicking pathological characteristics for disease modeling. 3.0-Mimicking structural characteristics, including complex features like osteons. 4.0-Advancing towards clinical translation, where bone/cartilage organoids can be applied in therapeutic contexts, including personalized medicine and large-scale tissue engineering

Additionally, accompanying the rapid advancement of AI typified by systems like GPT in recent years, AI has become increasingly influential in scientific research due to its exceptional ability to analyze complex datasets and extract meaningful patterns.^105,106^ It has also become a powerful tool in the field of tissue engineering and will also become a powerful tool in the optimization and functional enhancement of bone organoids in the future. AI algorithms can analyze complex datasets to identify optimal conditions for cell growth, differentiation, and organoid formation. This approach allows for the rapid screening of various biomaterials, growth factors, and environmental conditions, ultimately leading to the development of more efficient and effective protocols for organoid construction.^107^ AI screening is particularly valuable in identifying novel combinations of factors that promote the maturation and functionality of bone and cartilage organoids. The role of AI extends beyond construction, with high-throughput analysis and feedback loops enriching the development process (Fig. 7a). The iterative nature of this technology-a continuous loop of evaluation, data acquisition, and algorithm refinement-fuels organoid evolution, enhancing their resemblance to real tissues in both form and function.

Recently, AI has been employed to optimize the construction of organoids by improving the precision and efficiency of cell culture protocols. For instance, a study utilized machine learning algorithms to predict the optimal conditions for the differentiation of pluripotent stem cells into specific cell types, thereby enhancing the reproducibility and scalability of human midbrain organoid production.^108^ This technique and research route can be a reference scheme for the construction of bone organoids in the future. Furthermore, application of AI to predictive modeling of bone disease. AI models have been developed to optimize surgical planning for pedicle screw trajectories, considering bone mineral density and pull-out force. These models have shown superior performance in identifying optimized screw trajectories with higher bone mineral density and pull-out force compared to traditional methods, suggesting AI’s potential in improving clinical outcomes in bone-related procedures.^109^ Moreover, AI models have enhanced the detection of bone conditions, such as nasal bone fractures, by improving diagnostic accuracy and reducing variability among clinicians. The use of AI in these scenarios ensures more consistent and reliable interpretations, which can be crucial for the accurate development and assessment of bone organoids.^110^ These capabilities are now set to make significant impacts in the field of bone/cartilage organoid research, opening promising prospects for both regenerative medicine and the study of disease.

Through the synthesis of AI and state-of-the-art biofabrication techniques like bioprinting, a holistic and multi-faceted approach to organoid development is forged. This convergence is driving the field towards nuanced representations of bone and cartilage pathophysiology, unlocking new possibilities in disease modeling, pharmaceutical research, and beyond. As research and technological innovation proceed hand in hand, organoid technology stands on the cusp of realizing its full potential in biomedicine, marking a new epoch in the understanding and treatment of complex tissue disorders.

Meanwhile, we predict the iterative trends in the development of bone/cartilage organoids, roughly divided into four stages (Fig. 7b). Stage 1.0: Mimicking physiological characteristics focuses on accurately replicating the basic physiological properties of bone and cartilage tissues, including similar cell types, ECM components, and biological functions. Stage 2.0: Mimicking pathological characteristics involves introducing pathological conditions to study disease processes and responses to treatments. This is crucial for understanding how diseases such as osteoporosis or arthritis affect bone and cartilage tissues at the cellular and molecular levels. Stage 3.0: Mimicking structural characteristics aims to replicate the complex three-dimensional architecture of bone and cartilage tissues. This includes developing organoids with precise structural features such as osteons and cartilage layers, which are essential for studying the mechanical properties and functional integration of these tissues. Stage 4.0: Clinical translational applications focus on translating these advanced organoid models into clinical applications, including drug testing, personalized medicine, and regenerative therapies, thereby bridging the gap between laboratory research and real-world medical treatments.

Looking forward, there are exciting prospects for bone/cartilage organoids, thanks to advances in organoid techniques and microfluidic organ-on-a-chip technology. These developments could lead to a more dynamic representation of bone physiology, accounting for biomechanical stresses and the complex interactions within the bone microenvironment. Furthermore, gene-editing tools like CRISPR/Cas9 open new avenues for modeling bone disorders and drug development, allowing for the creation of personalized or disease-specific bone organoids. This could significantly enhance our understanding of tissue physiology and disease mechanisms. These organoids hold immense potential for modeling bone/cartilage development and disease, drug testing, and therapeutic applications. Continued research and technological advancements will undoubtedly bring us closer to fully realizing the potential of bone/cartilage organoid technology in biomedical engineering and regenerative medicine.
